# Fatigued individuals show increased conformity in virtual meetings

**DOI:** 10.1038/s41598-024-69786-6

**Published:** 2024-08-13

**Authors:** Lisa Masjutin, Anne Bangemann, Leonie Reimann, Günter W. Maier

**Affiliations:** Department of Psychology, Work and Organizational Psychology, Bielefeld University, Universitätsstr. 25, 33615 Bielefeld, Germany

**Keywords:** Psychology, Fatigue

## Abstract

Virtual meetings are widespread in organizations despite being perceived as fatiguing; a phenomenon also known as Zoom fatigue. Research suggests that Zoom fatigue is stronger when the camera is on, potentially influencing individuals to conform to majority opinions during professional online meetings. Two preregistered studies were conducted to explore the relationships between camera use, Zoom fatigue, social presence, and conformity. Study 1 involved 287 participants describing a professional online meeting in terms of the content discussed as well as the study variables. Study 2 involved 64 participants in an experimentally manipulated online meeting (camera on vs. off), focusing on a personnel selection task. We measured how many times participants changed their answers to match the majority and how this was related to Zoom fatigue and self-reported conformity. Results from both studies indicated that camera use was not related to either conformity or Zoom fatigue. Despite not finding the presumed mediation effect, the studies showed a clear link between fatigue and conformity. The results explain the emergence of conformity in online meetings and provide practical information for the design of video conferences.

## Introduction

Imagine Mary, who is working in a tech company. She works remotely as part of the company’s marketing team. Her job includes many virtual meetings in which new marketing strategies are discussed. Those meetings are often very long and exhausting. It is difficult to come to an agreement, and sometimes, no decision is made because of a lack of unanimity. Mary knows that there is an easy way to escape those exhausting discussions: Just say “yes” and agree with the majority and the discussion will find an end.

When decisions are made through group discussions, there is a risk of conformity. Conformity refers to the tendency of individuals to align their answer with the majority. While some level of conformity may be helpful for quick decision making, it can also lead to people failing to contribute their relevant knowledge during group discussions and, thus, reduce decision quality; individuals might even act against their morals because of conformity^1^. Conformity is a psychological phenomenon that has been extensively studied in face-to-face discussions^2,3^, but many questions about conformity in virtual settings are still unanswered.

Due to the high prevalence of virtual meetings in organizations, many decisions are made virtually instead of face-to-face. Despite the physical distance and lack of social cues, videoconferencing has the potential of creating social influence and thus inducing conformity^1,4^. The occurrence of conformity in virtual meetings is often explained through social presence, that is, the feeling of being with one another in networked environments^5^. According to this view, conformity arises when individuals feel the presence of others and perceive social cues. The probability of conforming to a majority in virtual meetings is boosted by the feeling of social presence if there is more interactivity or the use of live videos instead of pictures^4,6^.

Research has shown that individuals feel exhausted after participating in a virtual meeting^7–9^, especially if the camera has been switched on^10^. Due to Zoom’s dominant market position, the term “Zoom fatigue” has been coined for describing the feeling of exhaustion after videoconferencing. The consequences of fatigue during virtual meetings are unclear: A 4-week field experiment suggested that Zoom fatigue might hinder employee engagement^10^; however, associations with group decision making are still unknown. We argue that Zoom fatigue is also linked to conformity because highly fatigued individuals might be more likely to go along with the majority as it can be very exhausting to present one's own minority opinion.

**Figure 1:**
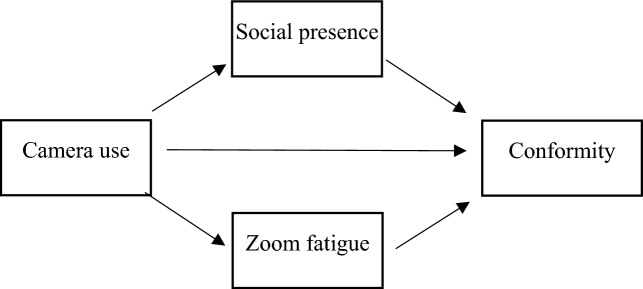
Theoretical framework.

In sum, the goal of the present work is to promote a better understanding of the extent to which camera use in virtual meetings induces conformity and which mechanisms mediate this relationship. We offer the mediator variables of Zoom fatigue and social presence to explain the relationship between camera use and conformity. We test our theoretical framework (see Fig. 1) with a multi-methods approach comprised of a field survey as well as an experiment. As such, we contribute to meeting science by comparing alternative explanations for the emergence of conformity in virtual meetings. Given the fatigue-inducing properties of virtual meetings, we propose Zoom fatigue as a novel mechanism that explains the reason individuals conform to the majority in group discussions.

### Conformity in virtual meetings

Many organizations have shifted from face-to-face meetings to virtual meetings using digital communication tools. There are many reasons for this. First, the COVID-19 pandemic necessitated remote work in many workplaces^11^. Thus, working remotely became more accessible and widespread for many employees. Today, employees are often not on site and virtual meetings ensure that they can still participate. Second, globalization has made virtual meetings a necessity to include international employees^12^. Third, virtual meetings use collaboration tools, such as digital whiteboards or voting tools, which facilitate the ease of reaching meeting goals, as they are often integrated in videoconference software. If the meeting goal is to design a new product, a collaborative online whiteboard can be used to jointly draw and integrate ideas using a shared visual workspace^13^. Fourth, greater time efficiency is expected. To take part in a virtual meeting, employees only have to click on the "Join meeting" button and do not have to change their locations. As a result, it is possible to switch meetings quickly as well as to quickly switch to other activities.

As discussed in the literature, organizational meetings have different purposes. In addition to information sharing, decision making is often cited as a reason to meet^14^. When group discussions are used for decision making, there is a risk of conformity: The social phenomenon of conformity describes the tendency of individuals to align their answers with the majority^2^. While this effect has been extensively researched in face-to-face interactions^3,15^, there are only a few studies that have investigated conformity in digital communications^16^. Cinnirella and Green compared face-to-face settings to computer-mediated settings and found conformity effects in both groups, with the face-to-face condition achieving higher conformity rates^17^. In their experiment, the computer-mediated condition was described as a chat-based interaction with no social cues other than text messages. The finding of lower conformity rates in computer-mediated interaction was explained by reduced social cues and, thus, less social presence. In a similar manner, another study on social presence and online conformity found higher conformity rates when participants could see live-videos of each other compared to only seeing a picture^4^.

Cinnirella and Green also presented another theoretical approach that would predict a different outcome^17^: The social identity model of deindividuation effects (SIDE^18^). The SIDE model suggests that conformity is influenced by the salience of social identity. When group membership is perceived as important, it is more likely that individuals conform to the group norms. Furthermore, the model predicts that in anonymous group situations, individuals might feel reduced self-awareness and a greater sense of social identity, which increases the risk of conformity. Huang and Li used this approach in a meta-analysis in which they summarized 13 articles on conformity in online contexts^16^. They found a positive relationship between anonymity and conformity, and this relationship was even stronger for visual anonymity, that is, not seeing your interaction partners. According to the SIDE model, we would expect the conformity rates to be higher when the cameras are switched off because visual anonymity would be higher.

The theoretical approaches seem to be in conflict with regard to the expected outcome for conformity in computer-mediated settings. On the one hand, switching on the cameras might cause higher conformity rates because social presence and accountability are higher. On the other hand, according to the SIDE model, camera use could reduce conformity as visual anonymity does not exist. However, inability to see your other group members will only increase anonymity in new groups in which the other members are unknown. In a work context where virtual meetings take place with colleagues, supervisors, or clients, the other participants are usually known, so visibility should not affect group anonymity. Moreover, the SIDE model predicts higher conformity rates when the salience of social identity is higher. According to this model, switching on the cameras might lead to greater visual presence of the group, increasing the salience of social identity. Group norms and expectations become more important, and participants will become more likely to adhere to group opinions; in other words, conforming to the majority. Based on the presented argumentation, we derive the following hypothesis:

Hypothesis 1: Switching on the cameras during virtual meetings increases conformity.

### Fatigue and social presence in virtual meetings

Interest in the effects of virtual meetings has increased since 2020 due to the drastic increase in remote work. Many users report pronounced feelings of exhaustion and fatigue after participating in videoconferences^19,20^. Before the topic sparked interest among scholars, it was already being discussed by the general public. Numerous news outlets have covered this phenomenon: In the Harvard Business Review, five tips on “how to combat Zoom fatigue” were given, and a BBC article presented reasons why “Zoom calls drain your energy”^21,22^. As a first theoretical approach on videoconference fatigue, Bailenson coined the term “Zoom fatigue” and theorized nonverbal overload as a potential cause for the occurrence of fatigue as a result of videoconferencing^7^. Nonverbal overload in videoconferencing refers to a situation in which there is an excessive number of nonverbal cues, such as facial expressions, body language, gestures, and eye contact. Bailenson provided four potential reasons for nonverbal overload. First, videoconferences are fatiguing due to eye gaze from a close distance. Compared to a real conference room where most participants sit distanced from each other and real eye contact is rarely made, videoconferences give the impression that all participants are looking at you from close range. Second, videoconferencing is associated with higher levels of cognitive load since sending and interpreting non-verbal signals in videoconferences is perceived as very stressful. Third, the presence of an all-day mirror in the form of the video self-view is argued to be fatiguing as well. Although most videoconferencing software offers a feature to hide the self-view, it is very rarely used. The effects of the self-view are explained by higher self-awareness, which leads to increased self-evaluation and social comparison. According to self-awareness theory^23^, when individuals see themselves, they become more attuned to their behaviors and appearance, often resulting in critical self-assessment and negative affect. Lastly, Bailenson mentioned the lack of mobility. Cameras have a fixed point of view in which the user has to be located for the duration of the meeting. In face-to-face meetings, people tend to be moving more. The suppression of movement during meetings can be fatiguing for individuals. The four theoretical mechanisms of nonverbal overload are associated to Zoom fatigue measured with the Zoom Exhaustion and Fatigue Scale^19,24^.

Most of the newly proposed mechanisms of nonverbal overload are directly linked to camera usage. For example, if the cameras are switched off, there will be no direct gaze from a close distance by the other participants. The cognitive load will be lower because no capacity to send exaggerated nonverbal cues, such as strong nodding, is required. The all-day mirror will disappear, and mobility might increase as there is no longer a fixed area to which the camera is directed. Shockley et al.^10^ empirically investigated the relationship between camera use and fatigue. They conducted a 4-week field experiment and manipulated camera use. They found that camera use was positively related to daily fatigue, while daily fatigue was negatively related to voice and engagement during meetings.

Hypothesis 2a: The effect of camera use on conformity is mediated by Zoom fatigue.

Switching on the camera in virtual meetings can also have positive effects. For example, it increases the feeling of being together in a virtual setting. This can be explained by the notion of social presence^5^. Initially, social presence theory was referred to as the relative salience of another in telecommunication, with each medium bringing its own degree of social presence^25^. More recent definitions consider other constructs besides perceived salience^26^ and distance themselves from technological determinism^27^. Perceived actorhood, co-location/non-mediation, understanding, association, medium sociability, and involvement as well as salience are among the relevant constituents of social presence^26^. More recent approaches to social presence suggest that social presence is determined by two key drivers, namely by the technology itself and by social and individual factors, such as personality, motivation, and context^27^.

In brief, social presence is defined by the feeling of being with other individuals and forgetting the mediation through media^28^. Switching on the camera during a virtual meeting might increase the feeling of being with other individuals for three reasons. First, if everyone in the conference switches the camera on, other participants not only can be heard but also seen. As an effect, the interaction is perceived as if the other participants were physically co-located, even though they are separated by technology. Second, camera usage could make the participants forget or downplay the mediation through media, as it simulates face-to-face interaction by creating an immersive experience. Lastly, camera usage can increase accountability. Knowing that others can see you can enhance a sense of accountability during the meeting. Kushlev and Epstein-Shuman^29^ observed that university students were more likely to stay engaged and actively participate when their camera was switched on during online classes. Based on the presented argumentation, we hypothesized that:

Hypothesis 2b: The effect of camera use on conformity is mediated social presence.

Although conformity in virtual meetings has already been investigated in some studies^1,4,6,30^, the mediating mechanisms are still unclear. The existing theoretical approaches on conformity in computer-mediated interactions are not sufficient to explain conformity in virtual meetings. In addition to social cues and social presence, we propose a novel working mechanism for the occurrence of conformity in virtual meetings. Until our studies, the fatigue-inducing properties, which are a crucial characteristic of videoconferencing, have not been taken into account. We propose a parallel working mechanism to social presence: Zoom fatigue caused by videoconferencing might make individuals more likely to conform to a majority. Fatigue leads to impaired cognitive control and affects cognitive task performance^31^. When fatigued, cognitive resources are reduced, including attention, self-control, and decision-making abilities. Moreover, a recent study in which virtual and face-to-face meetings were compared revealed that participating in virtual meetings resulted in passive fatigue, which, in turn, resulted in lower cognitive performance^32^. Fatigued individuals might lack the mental energy to thoroughly process information. As a result, they may conserve cognitive resources by relying on the answers of the majority as a shortcut. Another recent study found that Zoom fatigue was negatively associated with social connection^20^. This means that highly fatigued participants reported that they felt less socially connected to others in their meeting. We suppose that individuals might want to compensate for this lack of social connection by conforming to the group. Our assumption can be explained theoretically with the temporal need-threat model^33^. The need-threat model posits possible consequences if fundamental needs such as the need for belonging are not fulfilled. When the social connection is low and the need for belonging is at risk, individuals may perform need fortification to increase their connection to others. According to the model, need fortification can be achieved by compliance and conformity to the group. Thus, we derive the following hypothesis:

Hypothesis 3: Zoom fatigue is related to conformity in virtual meetings.

### Self-reported conformity

There are numerous studies that measure conformity as behavior in experiments^3,15^. Conformity is either operationalized as giving the same (often obviously wrong) answer as the majority^2^ or changing the initial answer to the answer of the majority^34^. Experiments are often criticized for their lack of external validity^35^. Many settings in conformity experiments are very artificial and will not be found in organizations; for example, it is very unlikely to discuss the length of lines with a group of strangers as in the classic experiment of Asch^2^. For this reason, we aim to create a measure of conformity to investigate the phenomenon in the field. To our knowledge, there is no instrument to measure subjective conformity behavior from the point of view of the acting individual.

According to Deutsch and Gerard^36^, conformity is divided into normative and informational conformity. Normative and informational conformity are driven by distinct motivations. Normative social influence is defined as an influence to conform to the positive expectations of another, while informational social influence can be defined as an influence to accept information received from another as evidence of reality. Informational and normative conformity can be considered separate processes^37^. Based on the model of Deutsch and Gerard^36^, we aim to develop a measurement instrument for self-reported conformity.

RQ1: Can conformity be measured in self-report?

RQ2: Do the effects differ for normative and informational conformity?

## Method

We conducted two studies. In study 1, we chose a cross-sectional design in which 287 participants were asked to describe one or two virtual meetings that included a group discussion. After describing this meeting, they had to answer questions about meeting characteristics, fatigue, social presence, and conformity. Study 1 was conducted to collect field data of real meetings and to test our theoretical model cross-sectionally.

Study 2 was an online experiment. We collected data on 64 individuals that participated in a virtual meeting with four to eight individuals. During this experiment, they were asked to compare two applicants for a job and choose the better qualified candidate. The answers given by the other participants were presented and the opportunity for the individual to change the answer was given. Conformity was measured by answer change. After the experimental procedure, participants filled in a questionnaire about fatigue, social presence, normative and informational conformity, and a manipulation check. Study 2 was conducted to manipulate camera use and to test causal effects of camera use on fatigue, social presence and conformity.

In the following chapter, the two studies are more thoroughly described.

### Transparency and openness

Prior to data collection, both studies were preregistered on AsPredicted. (See preregistration for study 1: https://aspredicted.org/blind.php?x=CL4_MSQ; study 2: https://aspredicted.org/blind.php?x=JRV_PG8). We changed the wording and the order of our hypotheses to be more concise. For clarification, H1 corresponds to H1 in preregistration 2. H2a + b corresponds to H1 in Preregistration 1 and to H2 + H3 in Preregistration 2. H3 corresponds to H2 in preregistration 1.

Both studies were performed in accordance with the Declaration of Helsinki and accepted by the university’s ethics committee (No 2022–255 and 2023–042). Informed consent was obtained from all participants before they took part in our studies. In addition, participants in study 2 were able to withdraw their consent after the debriefing about our experimental manipulation.

### Study 1

#### Research design and procedure

**Figure 2:**
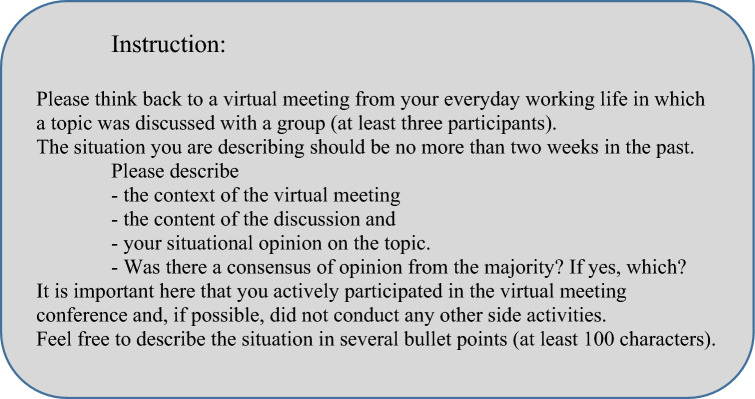
Instruction for study 1(translated from German).

For the first study, we chose a cross-sectional design. Data was collected in an online survey in December 2022. The procedure was as follows: Participants were informed of the content, survey duration, data protection regulations, and compensation. After consent, questions were asked relative to the participation requirements so that participants who did not meet the requirements could be filtered out. The exclusion criteria were: (a) working time of less than 17.5 h a week; (b) no use of virtual meetings at work; (c) an employment period of less than 6 months; and (d) pre-existing conditions that affect fatigue (e.g., neurological conditions). The survey started with demographic questions on age, gender, and job title. Afterwards, participants were asked to describe a virtual meeting in which a discussion was held. The instructions for this task can be found in Fig. 2.

After answering the questions, participants had the opportunity to describe another situation. This step could be skipped if the participant did not remember a second situation. We asked for a second situation because we wanted to increase the number of meetings in our dataset and compare meetings within subjects. Only 82 (28.6%) of the participants described a second meeting, and we decided to exclude the data from our analyses as we could not gather enough to make intraindividual comparisons. For the description of the first situation, participants used an average of *M* = 283.4 (*SD* = 153.4) characters. This demonstrates that our participants were appropriately engaged with the task, as they on average wrote more than twice as many characters as required.

Scholars report a significant increase in inattentiveness of survey respondents in online convenience survey samples^38^. Three attention checks were used in the survey to detect possible inattentive participants. The attention checks were in the beginning, in the middle, and at the end of the survey. One example item was, “We test your attention. Please select the option ‘Fully agree’ here.”

Finally, after participating in the survey, participants were asked to consent to the use of the data. Mean duration was 24 min (median = 10.2 min) and participants who completed the survey were compensated with 4€.

#### Participants

Participants were recruited on clickworker.de, a German crowdsourcing platform. As the targeted sample size was *N* = 300, we requested 350 adult clickworkers from Germany, Austria, and Switzerland and 314 of those 350 observations were considered completed. Twenty-seven participants had to be excluded from our dataset because at least one attention check was failed (*N* = 16, 59.3% of exclusions), no group discussion was described (*N* = 6, 22.2% of exclusions), consent for data usage was denied (*N* = 3, 11.1% of exclusions), or the questions were answered in English instead of German (*N* = 2, 6.8% of exclusions).

The final sample consisted of *N* = 287 working (at least 17.5 h per week) people, of which 38.3% were female (*N* = 110), 61.0% were male (*N* = 175), and 0.7% non-binary (*N* = 2). Participants’ ages ranged between 19 and 69 years (*M* = 39, *SD* = 11.18). Most of the participants worked in economic/administrative professions (*N* = 121; 42.1%), followed by research and education (*N* = 28; 9.8%) and information technology (*N* = 27; 9.4%). The most frequently used tools for virtual meetings were Zoom (*N* = 113; 39.4%) and Microsoft Teams (*N* = 109; 38.0%).

### Study 2

#### Research design and procedure

We conducted an experiment to compare the effects of camera use in virtual meetings. Data collection started in April 2023 and ended in May 2023. The procedure was as follows: Participants were recruited through social media and paper-based advertisements in the university building. In the recruitment text, we presented the cover story that our study is about hiring decisions and how different people make decisions. As an incentive for taking part in the experiment, a gift voucher of five euros was offered. For every experimental trial, we recruited four to eight participants. We decided to vary the group size between four to eight, as those are realistic numbers for participants in work meetings.

**Figure 3:**
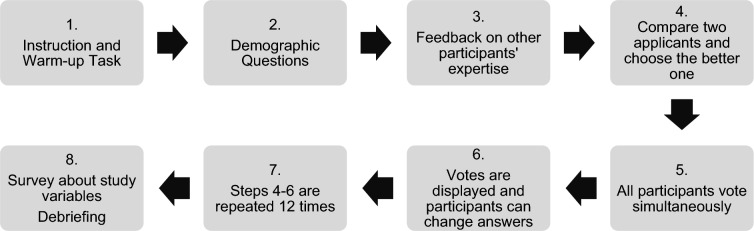
Sequence of study 2.

The experiment took place via Zoom. The participants registered with an e-mail address and were sent a Zoom link in advance. The sequence of our experiment is shown in Fig. 3.

**Figure 4:**
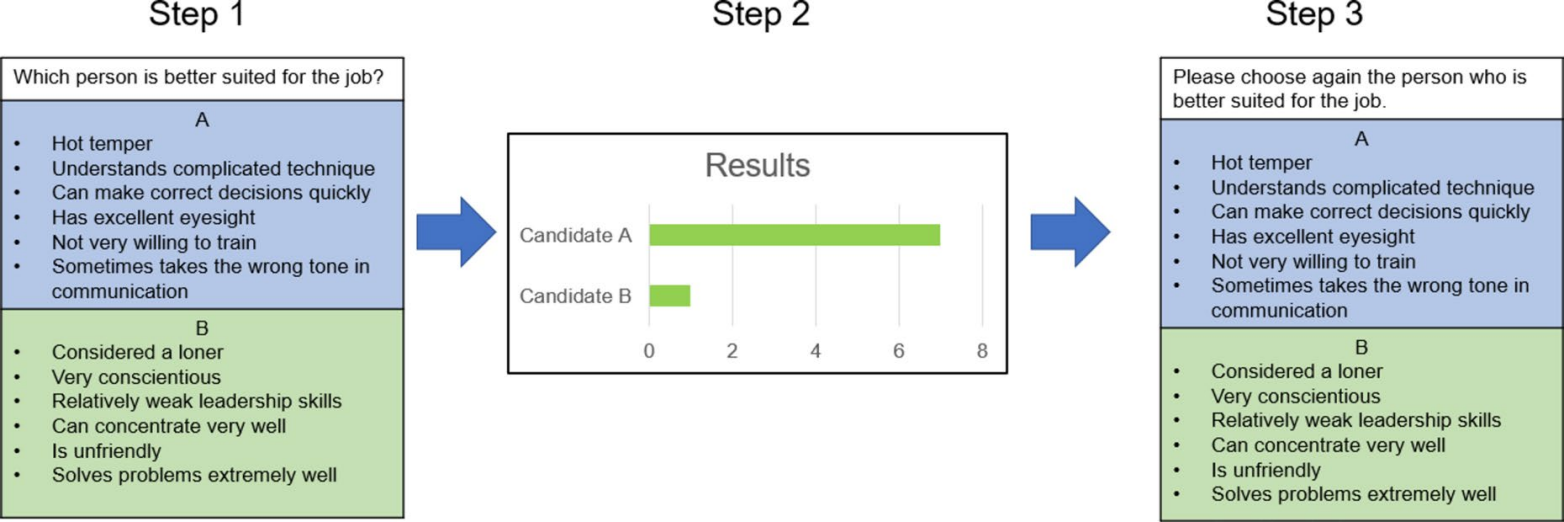
Example for a critical run (translated from German). This figure demonstrates a prototypical critical run in study 2. In this example, the participant picked candidate B. Our manipulated results (step 2) show that the participant is the only one who voted for candidate B, while all seven other participants voted for candidate A.

At the beginning of one experimental trial, all participants were either instructed to turn the camera on or to turn it off. Before the experiment started, a warm-up task was performed to enhance the participants’ group identity. The warm-up task was to find as many commonalities as possible in three minutes. Afterwards, the participants were asked to use a second device (e.g., their smartphone) to answer demographic questions. A second device was to be used to ensure that participants would not minimize the videoconference window. We asked whether the participants had experience in personnel selection. Regardless of the answers, every participant received the feedback that the other participants were very experienced in this area. This was done to raise the perceived competence and trustworthiness of the other participants. The twelve experimental runs then began. For one experimental run, participants were asked to compare two applicants for a job as a long-distance pilot and to choose the more qualified candidate. The applicants were described with three positive and three negative characteristics in random order to create an ambiguous task. The description of the candidates was derived from a study on group decision making^39^. After their decision, the manipulated decisions of the other participants were shown, and participants were asked to make their decision again. Out of the twelve experimental runs, participants saw eight critical and four distractor runs. During a critical run, the displayed answers were manipulated in such a way that the participant was always alone in the minority because the other participants had unanimously voted for the other applicant. An example of a critical run can be found in Fig. 4. For the distractor runs, the displayed votes were mixed without a clear majority.

After the twelve experimental runs, the experimenter thanked the subjects for their participation and asked them to complete the questionnaire on their own. In addition to the study variables, we asked if the participants had any comments about the study. At the end of the questionnaire, a debriefing about the true aim of our study was given and participants were asked to consent to data processing. The duration of the experiment was approximately 30 min.

#### Participants

Eighty subjects were recruited for our experiment. Eleven subjects did not complete the experiment until the end (for example, because of technical difficulties) and five had to be excluded from our final sample because they commented that they saw through our manipulation and doubted that the displayed votes by the other users were real. In the end, we collected 64 complete datasets.

The final sample consisted of 23 male and 40 female participants. One participant did not provide information on gender. Participants were between 20 and 72 years old (*M* = 43; *SD* = 14.4) and most were working (*N* = 56) or studying (*N* = 7). A total of 31 participants stated that they had experience in personnel selection, while 33 said they did not.

### Measures

The two studies used mostly equal measures. Any difference is stated in this section. If there was no German translation, the items were translated into German using the collaborative and iterative translation technique^40^. For study 1, participants were asked to recall the described meeting and to describe how they felt immediately afterward. For study two, participants were asked to think about the just-completed videoconference and to describe their feelings right now.

In study 1, camera use, hypothesized as an independent variable, was measured with the item “How was the camera function of your device set during the videoconference described?” (Responses: 0 = The camera was switched off for more than 50% of the videoconference; 1 = The camera was switched on for more than 50% of the videoconference). Additionally, we asked if other participants in the videoconference had their camera switched on. We used the item “Were you able to see the other participants during the video conference?” (Responses: 0 = No, less than 50% of the videoconference; 1 = Yes, more than 50% of the videoconference). In study 2, all participants were either instructed to turn the camera on or to turn it off. To check whether our manipulation was successful, we asked the participants if their cameras were switched on during the experiment (0 = No; 1 = Yes) Furthermore, we asked whether the participants could see the other participants (0 = No; 1 = Yes; 2 = Partly).

Zoom fatigue, which was hypothesized as a mediating variable, was measured using the Zoom Exhaustion and Fatigue Scale^24^. Responses were given on a five-point Likert scale (α = 0.82 in study 1, α = 0.88 in study 2). A sample item is: “After the described videoconference, I felt tired.” In study 2, Zoom fatigue was also measured with the item “Right now, I feel fatigued.” by Shockley et al.^10^.

Social presence, another mediating variable, was measured with eight items from Jang and Choi^28^. Responses were given on a five-point Likert scale (α = 0.95/0.85). A sample item is: “I felt like I was in the same room with other participants.”

#### Self-reported conformity scale

To our knowledge, there is no valid and reliable instrument to measure conformity. To test our hypotheses in study 1, we created the self-reported conformity scale consisting of two dimensions: informational and normative conformity. The content of the items was derived according to the definitions of Deutsch and Gerard^36^, and the content was validated according to Colquitt^41^. A sample item for normative conformity is: “I felt more comfortable with agreeing with the majority”; and for informational conformity is: “I trusted the majority in the discussion as they were more knowledgeable about the subject” (see Supplementary Table S1 for a full list of our items). In study 2, we measured conformity as a behavioral variable: If a participant changed their answer during a critical trial, this was seen as conformity. This approach to measuring conformity has been used in several previous experiments (e.g., ^6,30,34,42^). In addition, we used the self-reported conformity scale in study two to validate it in a second sample.

##### Exploratory factor analysis (EFA)

In order to determine the factor structure of the newly developed self-reported conformity scale, we conducted an EFA on the data of study 1. Results of Kaiser–Meyer–Olkin test (KMO-coefficient = 0.901) and Bartlett’s test for sphericity (χ^2^ (78) = 1738; *p* < 0.001) confirm the suitability of our data to perform an EFA.

To examine the structure of the scale, we performed EFA with maximum likelihood extraction method and varimax rotation. Our supposed two-factor structure of conformity was supported by the eigenvalues, as two factors had eigenvalues greater than 1 and explained 51,2% of the total variance (see Supplemental Fig. S2 for scree plot of EFA). Factor loading and variance explained results from the EFA are shown in Supplemental Table S3.

##### Confirmatory factor analysis (CFA)

We continued our analyses with a CFA to test the construct validity of our newly developed scale. We assigned the items to the respective factors using our theoretical assumptions of conformity. The results of CFA are displayed in Table 1. We compared a unidimensional model in which all of the items are loading on one single factor to a two-dimensional model based on Deutsch and Gerard’s^36^ theoretical model on conformity. A chi-square difference test revealed a significantly better model fit for the two-factor solution (Δχ^2^(1) = 148.23, *p* < 0.001). Nevertheless, the fit indices (CFI = 0.82; RMSEA = 0.13) showed that the model fit could be improved. Based on the modification indices and on the factor loadings, we eliminated items to improve our model fit. We decided to keep a maximum of three items with the highest factor loadings per subscale for a second confirmatory factor analysis. Items with the lowest loadings were eliminated for further analyses; the respective fit indices of those models are displayed in the lower part of Table 1. The two-factor model of the six-item version shows an acceptable fit. Most fit indices reach the criteria of acceptable fit^43^. RMSEA alone does not reach the recommended threshold of below 0.06. However, it should be noted that RMSEA is less robust in models with small degrees of freedom^44^. As we could reach acceptable fit indices with our six-item version, we used it for the analyses.

**Table 1:** Confirmatory factor analysis results for the self-reported conformity scale.

Model	Χ^2^	df	Δχ^2^(Δdf)	TLI	CFI	RMSEA	SRMR
Study 1 (*N* = 287)							
One factor (13 items)	521.37	65		0.73	0.73	0.16	0.12
Two factors (13 items)	373.14	64	148.23 (1)	0.77	0.82	0.13	0.12
One factor (6 items)	112.48	9		0.73	0.84	0.20	0.10
Two factors (6 items)	35.27	8	77.21 (1)	0.92	0.96	0.11	0.06

χ^2^, chi-square fit index; df, degrees of freedom; TLI, Tucker–Lewis index; CFI, comparative fit index; RMSEA, root mean square error of approximation; SRMR, standardized root mean squared residual.

## Results

### Study 1

The descriptive statistics and intercorrelations of the variables under investigation are displayed in Table 2.

**Table 2:** Summary of intercorrelations, means and standard deviations for variables under investigation in study 1.

		*M*	*SD*	1	2	3	4	5	6	7	8	9
1	Camera use (own)	0.84	0.36	–								
2	Camera use (others)	0.93	0.26	0.504**	–							
3	Age	38.77	11.18	0.002	0.072	–						
4	Relevance	4.31	0.76	0.064	− 0.044	0.138*	–					
5	Zoom Fatigue	1.75	0.71	− 0.041	− 0.002	− 0.101	− 0.181**	*0.82*				
6	Social Presence	3.25	0.80	− 0.022	0.070	0.130*	0.265**	− 0.231**	*0.95*			
7	Norm. Conformity	2.20	0.82	− 0.078	− 0.002	− 0.220**	− 0.096	0.336**	0.083	*0.84*		
8	Inf. Conformity	2.72	0.77	− 0.016	− 0.005	− 0.163**	− 0.086	0.075	0.209**	0.484**	*0.73*	
9	Conformity (total)	2.40	0.72	− 0.057	0.001	− 0.225**	− 0.106	0.249**	0.164**	0.882**	0.839**	*0.82*

*N* = 287 *p < .05, **p < .01. Values in italic in diagonal are reliability coefficients. Camera use was coded 0 = no camera, 1 = camera. Camera use (own) indicates whether the study participants had their own camera switched on and camera use (others) indicates whether other participants in the virtual meeting were visible. We used the short six-item scale for conformity and the three-item subscales for normative and informational conformity.

#### Hypothesis testing

**Figure 5:**
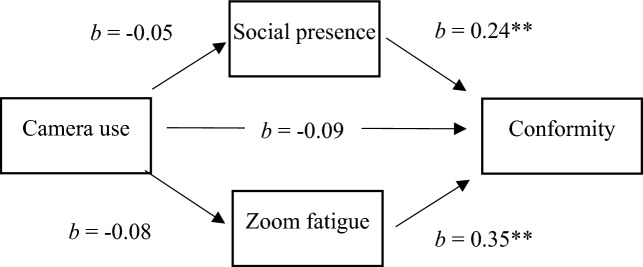
Results of mediation analyses for predicting self-reported conformity for study 1.

To test the hypotheses, we computed a mediation analysis using the macro PROCESS for SPSS^45^ with a categorical independent variable (PROCESS version 3.1, SPSS version 28). The results of the mediation analysis are displayed in Table 3 and Fig. 5.

**Table 3:** Mediation analyses for the relation of camera use and conformity, mediated by Zoom fatigue and social presence (study 1).

Antecedent	Consequent	Constant (SE)	*b*	*SE b*	*p*
Camera use	Social presence	3.31 (0.16)	− 0.05	0.13	0.708
Camera use	Zoom fatigue	1.84 (0.14)	− 0.08	0.12	0.487
Social presence	Conformity	1.09 (0.30)	0.24**	0.06	< 0.001
Zoom fatigue	Conformity	1.09 (0.30)	0.35**	0.042	< 0.001
Camera use	Conformity	1.09 (0.30)	− 0.09	0.67	0.486

*N* = 287. *p < .05, **p < .01. Camera use was coded 0 = no camera, 1 = camera.

To test hypothesis 1, we investigated the path from the independent variable camera use to the dependent variable self-reported conformity. Camera use did not relate to self-reported conformity (*b* =  − 0.09; *p* = 0.486). The direct effect is not significant; therefore, we reject hypothesis 1.

For hypothesis 2a and 2b, we relied on the confidence intervals of the relative indirect effects, which are the product of the effect of camera use on the mediator and the effect of the mediator on self-reported conformity. We used PROCESS with 5000 bootstrap samples to compute 95% confidence intervals for the relative indirect effects. If the confidence intervals did not include zero, the relative indirect effect was statistically significant and indicated mediation. Regarding H2a for the effect of camera use on self-reported conformity mediated by fatigue, the confidence interval for the indirect effect included zero (*95% CI* = [− 0.10; 0.59]), which means that there is no significant mediation. For the effect of camera use on self-reported conformity mediated by social presence (H2b), the confidence interval for the indirect effect included zero (*95% CI* = [− 0.08; 0.06]), which means that the effect of camera use on self-reported conformity is not mediated by social presence. In summary, the data does not support hypotheses 2a-b.

For hypothesis 3, we investigated the path from the mediator variable Zoom fatigue to the dependent variable self-reported conformity. Zoom fatigue is significantly related to self-reported conformity (*b* = 0.35; *p* < 0.001). The direct effect is significant, which supports hypothesis 3.

### Study 2

#### Missing data analysis

Five questionnaires had missing values. The Little chi-square statistic^46^ showed that the values were not missing completely at random (MCAR) χ^2^ (159) = 221, *p* < 0.001. MCAR means that the missing data are independent of both observed and unobserved data^47^, which our test results rejected. However, this does not exclude the possibility that the data are missing at random (MAR), where the probability of missingness is related to observed data but not the unobserved data. For this reason, we assume the values were MAR. This affected 21 out of 4608 values (0.456%) on 13 variables. The missing variables were filled in using random forest imputation. Random forest uses machine learning for data imputation and has been shown to be superior to other imputation methods across many datasets and heterogeneous data^48^. All further calculations were performed on the imputed data.

#### Manipulation check and descriptive statistics

To check whether our manipulation was successful, we asked the participants if their cameras were switched on during the experiment. This question was answered correctly by 100% of the participants. Furthermore, we asked whether the participants could see the other participants. In line with the experimental conditions, all participants reported being able to see or not see the other participants; only one person in the "camera on" condition reported being able to partially see the others.

During the critical tasks, 78% of the participants adapted their answers to the majority at least once, in distractor tasks only 33%. A Chi-Square test identified a significant relationship between type of task and answer change (Χ^2^ (1), = 25.602, *p* < 0.001.). Our manipulation of conformity was successful because participants were more likely to change their answer when they completed an experimental task (see Table 4).

**Table 4:** Contingency table of answer change and experimental task.

	Answer change	
	No	Yes
Distractor task	227 (29.55%)	29 (3.78%)
Experimental task	370 (48.18%)	142 (18.49%)

Sixty-four participants completed twelve tasks each, resulting in a total of 768 trials. Every participant completed eight experimental tasks and four distractor tasks.

The descriptive statistics and intercorrelations of the variables under investigation are displayed in Table 5.

**Table 5:** Summary of intercorrelations, means and standard deviations for variables under investigation in study 2*.*

		*M*	*SD*	1	2	3	4	5	6	7	8	9
1	Camera use	0.45	0.50	–								
2	Age	43.06	14.42	− 0.00	–							
3	Group size	7.02	1.21	− 0.19	0.13	–						
4	Zoom fatigue	1.36	0.41	− 0.02	0.13	0.09	*0.88*					
5	Social presence	2.55	0.89	0.11	− 0.17	− 0.13	0.06	*0.85*				
6	Norm. conformity	1.54	0.68	− 0.00	0.17	− 0.10	0.36**	0.21	*0.89*			
7	Inf. conformity	1.54	0.81	0.11	0.09	0.00	0.22	0.14	0.66**	*0.90*		
8	Conformity (total)	1.54	0.68	0.06	0.14	− 0.05	0.28*	0.17	0.85**	0.93**	*0.93*	
9	Answer change	2.22	2.04	0.06	− 0.03	− 0.06	0.36**	0.11	0.50**	0.45**	0.52**	–

*M* and *SD* are used to represent mean and standard deviation, respectively. *N* = 64. *p < .05, **p < .01. Values in italic in diagonal are reliability coefficients. Camera use was coded 0 = no camera, 1 = camera.

#### Hypothesis testing

**Figure 6:**
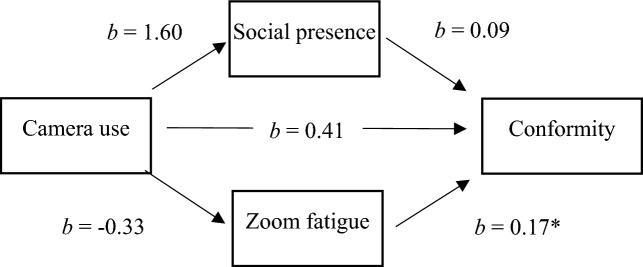
Results of mediation analysis for predicting self-reported conformity for study 2.

**Figure 7:**
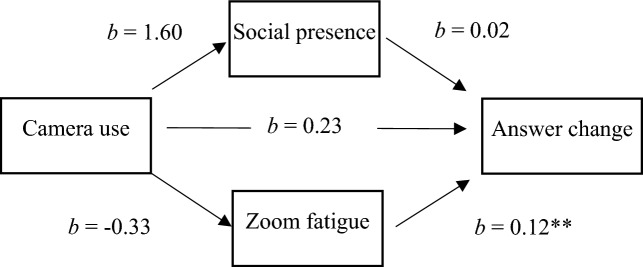
Results of mediation analysis for predicting answer change for study 2.

To test our hypotheses of study 2, we chose the same approach as for study 1: We conducted mediation analyses using the SPSS PROCESS Macro^45^. The results are displayed in Table 6 for the dependent variable answer change and in Table 7 for the dependent variable self-reported conformity. Similar to study 1, we could not find support for hypotheses 1 and 2a + b. Camera use had no effect on either answer change (*b* = 0.23; *p* = 0.635, H1) or on self-reported conformity (*b* = 0.41; *p* = 0.668, H1), and the indirect effects were not significant for either Zoom fatigue or social presence. As in study 1, we investigated the direct path from the mediator variable Zoom fatigue to the dependent variable self-reported conformity. We inspected both the self-reported conformity scale (see Fig. 6) and answer change (see Fig. 7) as dependent variables. For both variables, the direct effect from Zoom fatigue was significant (*b* = 0.17; *p* < 0.05 for self-reported conformity; (*b* = 0.12; *p* < 0.005 for answer change). This supports hypothesis 3.

**Table 6:** Mediation analyses for the relation of camera use and answer change, mediated by Zoom fatigue and social presence (Study 2).

Antecedent	Consequent	Constant (SE)	*b*	*SE b*	*p*
Camera use	Social presence	19.78 (1.20)	1.60	1.78	0.371
Camera use	Zoom fatigue	20.31 (1.03)	− 0.33	1.53	0.825
Social presence	Answer change	− 0.79 (1.10)	0.02	0.03	0.510
Zoom fatigue	Answer change	− 0.79 (1.10)	0.12*	0.04	0.004
Camera use	Answer change	− 0.79 (1.10)	0.23	0.49	0.635

*N* = 64. *p < .05. Camera use was coded 0 = no camera, 1 = camera.

**Table 7:** Mediation analyses for the relation of camera use and self-reported conformity, mediated by Zoom fatigue and social presence (study 2).

Antecedent	Consequent	Constant (SE)	*b*	*SE b*	*p*
Camera use	Social presence	19.78 (1.20)	1.60	1.78	0.371
Camera use	Zoom fatigue	20.31 (1.03)	− 0.33	1.53	0.825
Social presence	Conformity	3.16 (2.13)	0.09	0.07	0.178
Zoom fatigue	Conformity	3.16 (2.13)	0.17*	0.08	0.031
Camera use	Conformity	3.16 (2.13)	0.41	0.96	0.668

*N* = 64. *p < 0.05. Camera use was coded 0 = no camera, 1 = camera. Conformity was measured by the short 6-Item Conformity Scale.

#### Exploratory analyses

To investigate our research questions (RQ1 and RQ2), we further examined the correlation table (Table 5). We found a strong, significant correlation between answer change and self-reported conformity (*r* = 0.52, *p* < 0.01). This result addresses RQ1 and shows that our scale enables measuring conformity in self-report. For RQ2, we examined the correlations of normative and informational conformity in Table 5. Interestingly, normative conformity is correlated to Zoom fatigue (*r* = 0.36, *p* < 0.01), whereas informational conformity is not (*r* = 0.22, *p* > 0.05). This shows that fatigued individuals are more likely to follow the majority because of peer pressure and not because they accept the majority’s answer as true.

## Discussion

Our work is the first known attempt to investigate conformity in the context of virtual meetings by taking Zoom fatigue into account. By doing so, we contribute to theory formation as the mechanisms to explain how camera use affects conformity are unclear to date. In the following, we summarize the results of our studies, discuss theoretical implications and limitations, and suggest future research directions.

In study 1, we chose a cross-sectional design to investigate the interrelations of the variables of camera use, Zoom fatigue, social presence, and conformity. We hypothesized that the relation of camera use and conformity were mediated by Zoom fatigue and social presence. Based on the theory of nonverbal overload^7^, we expected a clear association between camera use and Zoom fatigue. Our hypotheses concerning camera use were not supported by our data. Nevertheless, our findings provide important insights into social processes during virtual meetings. The results of our first study show that social presence and Zoom fatigue are positively correlated with conformity. This means that highly fatigued and highly socially present individuals are more likely to adapt their answers to the majority.

Surprisingly, we did not find an association between camera use and Zoom fatigue. Our results imply that the supposed influence of camera use on fatigue is not as strong as expected. In contrast to experimental research^10^, we did not experimentally manipulate camera use in study 1. Many employees do not have the choice to switch their camera on or off in work meetings because there are often implicit or explicit norms regarding camera use in organizations. Furthermore, the findings on camera use and Zoom fatigue are not consistent. For example, camera use during online university classes did not increase student fatigue^29^. In addition, students predicted that switching on the camera would increase their fatigue, although this was not the case, showing that students might overestimate the disadvantages of camera use.

In study 2, we used an experimental approach to investigate the study variables. We manipulated camera use (camera on vs. camera off) and measured Zoom fatigue, social presence, and conformity. Conformity was measured as a behavioral variable (answer change) and as a self-report to validate our newly developed scale. Our results in study 2 were similar to the results in study 1. We could not find evidence for our hypotheses concerning camera use (H1 and H2a + b). Regardless of whether the camera was switched on or off, it did not affect the participants’ urge to conform to the majority (measured by self-report) or the tendency to adapt their answers to the majority (measured as a behavioral response). However, our findings complement existing research by showing that conformity occurs in virtual meetings and that it is high for ambiguous stimuli. When there was a clear majority, 78% of participants changed their answers to the majority at least once. This is consistent with previous findings that ambiguous stimuli lead to higher conformity^3,4^.

Nevertheless, we found an association between fatigue and conformity in both studies. This finding is particularly interesting as fatigue presents a new predictor for conformity. In former research, conformity was explained as a social phenomenon and as a product of social processes^2,3^. As new media arises and contexts change, we propose a novel theoretical approach for conformity. Individuals not only conform because of normative or informational influences^36^ but also might conform to the majority because of fatigue. In highly fatiguing situations, such as virtual meetings, conformity might be a shortcut to end the meeting and start recovery.

Although camera use is theorized to affect Zoom fatigue according to the theory on nonverbal overload^7^, we failed to find this association in two samples: In neither our cross-sectional survey nor in our experiment did we find a significant correlation between camera use and fatigue. Therefore, we cannot with a clear conscience promote camera use as the most important trigger for Zoom fatigue to the extent theorized by many researchers. To our knowledge, only one study experimentally manipulated camera use in virtual work meetings and found camera use to predict fatigue^10^. Other studies only investigated Zoom use with the camera on^49^, or found no significant correlation between camera use and Zoom fatigue^50^, or provided qualitative results for the association between camera use and Zoom fatigue^50,51^. Interestingly, Lübstorf et al.^51^ found the opposite of Shockley et al.^10^: Meeting leaders indicated that turning the camera off was stressful, especially when other meeting participants turned their cameras off and were not visible to the meeting leaders. As there are obvious inconsistencies concerning the assumed effect of camera use on Zoom fatigue, we call for further research to investigate it. Another experiment compared audio- and videoconferences and found that the availability of video did not enhance strain of call center agents^52^.

Even if camera use did not appear to be the reason for this, participants in our studies were still tired from attending the virtual meeting. We suggest three possible reasons for Zoom fatigue to occur besides camera usage. First, participating in virtual meetings means doing screen work. A high amount of screen work is associated with a higher risk of musculoskeletal symptoms^53^ and digital eye strain^54^. Digital eye strain is a condition with ocular symptoms arising due to prolonged exposure to digital devices. In addition to ocular symptoms such as dry or burning eyes, digital eye strain also includes non-ocular symptoms such as general fatigue^55^. One of the five dimensions of Zoom fatigue in the Zoom Fatigue and Exhaustion Scale is visual fatigue^24^. Regardless of whether the camera is switched on or off, virtual meetings require participants to focus on digital content for an extended period and the constant exposure can contribute to fatigue.

Second, technical challenges and distractions, such as poor internet connectivity or audio problems, can disrupt the flow of a virtual meeting. Dealing with these technical distractions can be frustrating and tiring. Participants may find themselves spending more time fixing technical issues than actively engaging in discussions.

Third, multitasking and task switching during virtual meetings can be a fatiguing aspect. During face-to-face meetings, it is not possible or considered rude to switch to another task, such as checking the phone or answering e-mails. However, during virtual meetings, secondary activities, such as checking your appointments and preparing for the next meetings, can be easily accomplished as participants are already sitting in front of their computers. Riedl^56^ argued that engaging in multiple unrelated activities while in a virtual meeting is a root cause of Zoom fatigue because it costs cognitive resources and increases the workload of individuals. Additionally, a recent meta-analysis on the biological effects of multitasking has found higher physiological stress responses during dual- and multi-tasking^57^.

To assess conformity in study 1, we developed the self-reported conformity scale. Conformity is a ubiquitous behavioral phenomenon that has been observed in a large number of studies^3,16^. Our work is the first to examine self-reported conformity (study 1) and to compare our measure of self-reported conformity to an actual behavioral measure (study 2). We found the two-factor structure of our self-reported conformity scale to be a fit to the data. As the scale is new and has only been tested on two samples, a further validation of the two-factor structure on another sample is yet to come.

We found self-reported conformity and answer change to be correlated in study 2. Participants who often changed their answers during the experimental trials were more likely to score a high value on the self-reported conformity scale. This shows that our scale is a valid measure for conformity and that participants are able to voice that they changed their answer either because of normative conformity or informational conformity.

### Limitations, recommendations for future research and practice

Even though we used two study designs and samples to investigate conformity and Zoom fatigue in virtual meetings, there are several limitations. First, we will discuss the limitations of studies 1 and 2 and then we will give further directions for research and practice.

For study 1, we opted for a cross-sectional design. As our study is the first to put conformity and Zoom fatigue in context, we chose a cross-sectional approach to shed the initial light on this relationship. However, the correlational nature of our data does not allow causal inferences. For this reason, we supplemented study 1 with an experiment and were able to replicate the significant relationship of Zoom fatigue and conformity. Further experiments on conformity in virtual meetings should follow. A follow-up experiment could directly manipulate the participants’ fatigue and investigate its effects on conformity. However, our study implies that camera use might not be suitable for manipulating fatigue, as it had no effect on our participants. Further experiments have to find other predictors of Zoom fatigue, for example, multitasking and task switching^56^, to effectively manipulate the construct.

In study 1, we asked participants to describe a situation in retrospective. They were asked to describe a situation that occurred no more than 2 weeks ago. It might be that the participants did not have a good memory of the situation and their fatigue. To ensure data quality, we asked the participants to describe the situation thoroughly (at least 100 characters). We also asked specific questions to bring the content of the virtual meeting back to mind. This description leads to the recall of the situation and puts the participants in their emotions and thoughts during the situation. Our procedure was based on the event reconstruction method^58^. This method was compared to traditional experience sampling methods and yielded similar results for affective outcomes, such as job satisfaction. Even though we did not collect the participants’ responses directly after the virtual meeting, we ensured valid results by providing detailed instructions, thereby promoting intensive engagement with the situation.

In study 2, participants had to use two devices: On one device, they participated in the virtual meeting via Zoom. Participants were asked to use a laptop or a desktop personal computer so that the videoconference could be seen on a larger screen. The survey items were presented on a second device, for example, their smartphone. Two devices were used to ensure that the participants would not minimize the Zoom application when answering the items on the personnel selection task. On the one hand, using two devices might have reduced the effects of videoconferencing. The Zoom fatigue-inducing properties, such as eye gaze at close distance or the all-day mirror, may have been less relevant because of this setup. Further research should investigate the possibility of reducing Zoom fatigue by implementing a second device. If this assumption is confirmed, second devices could be strategically used in virtual meetings to reduce participant fatigue and, thus, their tendency to conform to the majority. On the other hand, handling two devices at the same time might be considered multitasking and therefore be as stressful. As indicated by Riedl^56^, multitasking during virtual meetings is proposed as a root cause of fatigue.

It is also possible that our manipulation of conformity in study 2 was not successful. We used a personnel selection task. Participants were to indicate whether they preferred candidate A or B for the job of a long-distance pilot. After they gave their answers, we showed them the manipulated votes of the other participants. Similar approaches have been used in other conformity experiments^30,42^. In an open survey question at the end of the experiment, some participants expressed doubts that the votes displayed by others were genuine. Those were eliminated from our dataset. However, it is unknown how many participants did not voice their doubts but still were suspicious of our experimental setup. Allen^59^ indicated that individual participants reported doubts about the authenticity of the majority that they had not voiced right after the experiment. These doubts were revealed to the experimenter during random encounters after the experiment had been conducted. It might likewise be possible that our dataset holds data from doubtful participants. However, our manipulation check compared answer changes during distractor trials and critical trials and showed that it was more likely for participants to change their answers during critical trials. This shows that our manipulation was indeed successful, revealing that the majority had a clear effect on the participants. We also complemented our experiment with a field survey (study 1). In study 1, participants were asked to describe a virtual meeting from their workplace. This allowed us to analyze conformity in real environments without participants having doubts about artificial majorities.

One limitation of study 2 is that all participants were strangers to each other and were unlikely to interact again in the future. This context reduces the relevance of impression management compared to work meetings where participants often include supervisors, coworkers, and clients, and where there are established norms and regulations regarding camera use. In professional settings, individuals are more likely to engage in impression management to maintain their professional image and relationships. This difference could affect the generalizability of our findings to real-world work environments, where the stakes of impression management are higher. To address this limitation, future research could explore similar experiments in actual work settings, despite the inherent challenges of manipulating camera use in such environments.

We developed a novel, two-dimensional scale to measure conformity. This scale measures the participant’s tendency to conform to a majority for normative reasons (i.e., group pressure) and informational reasons (i.e., believing that the majorities’ answer is better). We found an acceptable model fit for a short six-item solution. Our scale was correlated to the behavioral response in study 2. Participants who often adapted their answer to the majority were more likely to score highly on the conformity scale. This finding supports the validity of our scale. However, the validity of the self-reported conformity needs to be replicated in further samples.

Our work is one of the first to investigate the effects of camera use in virtual meetings. In study 1, we asked participants if their own camera was switched on and if they could see the other participants. In study 2, we manipulated camera use, so that either all participants of the videoconference had their camera switched on or off. In that way, we did not differentiate between being seen (own camera use) and seeing others (other’s camera use). Future work should investigate differences between being seen and seeing others in virtual meetings. Furthermore, participants might switch their camera on or off after some time has passed, which we did not include in our studies.

## Conclusion

Important decisions, such as the selection of job applicants, are made in virtual meetings. The question of whether participants can freely voice their opinions or are more subject to conformity and whether this is related to their fatigue was investigated in this work. In summary, we conducted two studies to investigate the associations between camera use, Zoom fatigue, social presence, and conformity. We developed a scale for self-reported conformity that measures an individual’s subjective urge to conform to a majority in retrospective. Our self-reported measure is significantly related to the actual answer change in an experiment. We did not find camera use to be related to Zoom fatigue, social presence, or conformity. We could not replicate the proposed negative effects of camera use in virtual meetings and therefore call for future research to investigate predictors of Zoom fatigue other than camera use. In both studies, we found that fatigued individuals were more likely to conform to the majority. This finding broadens the perspective on conformity and reveals practical implications for the implementation of group discussions in virtual meetings.

### Supplementary Information

Supplementary Information.

## Data Availability

The datasets analyzed during the current study are available from the corresponding author on reasonable request.
