# Supplementary material for: Fatigued individuals show increased conformity in virtual meetings
Source: Sci Rep. 2024 Aug 13;14:18807. doi: 10.1038/s41598-024-69786-6 (PMC11322159; doi:10.1038/s41598-024-69786-6)
Supplement: Supplementary file 1 — Supplementary Information. [file 41598_2024_69786_MOESM1_ESM.docx]

| Item | German Version (original)  *English Version (translated)* | *M* | *SD* | *ITC* |
| --- | --- | --- | --- | --- |
| Kon_N_1 | Ich hatte das Gefühl, mit den anderen der Gruppe übereinstimmen zu wollen.  *I felt like I wanted to agree with the others in the group.* | 3.01 | 1.12 | .306 |
| Kon_N_2 | Ich hatte das Gefühl, von der Mehrheit unter Druck gesetzt zu werden.  *I felt like I was being pressurized by the majority.* | 1.88 | 1.06 | .567 |
| Kon_N_3 | Ich konnte meine echte Meinung nicht äußern, da ich Gruppendruck verspürt habe.  *I couldn't express my real opinion because I felt peer pressure.* | 1.94 | 1.12 | .611 |
| Kon_N_4 | Ich habe mich damit wohler gefühlt, der Mehrheit zuzustimmen.  *I felt more comfortable agreeing with the majority.* | 2.64 | 1.18 | .687 |
| Kon_N_5 | Ich hatte Angst, aufgrund meiner Meinung von den anderen abgelehnt zu werden.  *I was afraid of being rejected by others because of my opinion.* | 1.90 | 1.12 | .602 |
| Kon_N_6** | Ich habe es bevorzugt, meine Äußerung an die Erwartungen der anderen anzupassen.  *I preferred to adapt what I said to the expectations of others.* | 2.11 | 1.14 | .714 |
| Kon_N_7** | Ich habe Druck von der Mehrheit gespürt, meine Meinung zu ändern.  *I felt pressure from the majority to change my opinion.* | 1.96 | 1.18 | .625 |
| Kon_N_8** | Ich habe mich in der Diskussion der Mehrheit angeschlossen, da es die anderen von mir erwartet haben.  *I joined the majority in the discussion because the others expected me to do so.* | 2.17 | 1.23 | .692 |
| Kon_I_1 | Ich hatte das Gefühl, dass die anderen bessere Argumente als ich hatten.  *I had the feeling that the others had better arguments than me.* | 2.38 | 1.05 | .455 |
| Kon_I_2 | Ich habe dem Urteil der Mehrheit vertraut.  *I trusted the judgement of the majority.* | 3.15 | 1.05 | .374 |
| Kon_I_3** | Ich habe der Mehrheit in der Diskussion vertraut, da sie sich besser mit dem Thema auskannten.  *I trusted the majority in the discussion because they were more familiar with the topic.* | 2.38 | 1.17 | .629 |
| Kon_I_4** | Die Sichtweise der Mehrheit ist mir logischer vorgekommen.  *The majority's view seemed more logical to me.* | 2.96 | 1.01 | .408 |
| Kon_I_5** | Ich habe mich auf das Urteil der anderen verlassen.  *I relied on the judgment of others* | 2.71 | 1.14 | .566 |

**Table S1.** Full list of our items of the Self-reported Conformity Scale. Items were rated on scale from 1 (do not agree at all) to 5 (fully agree). Means and standard deviations are taken from study 1. ITC = Item Total Correlation

**final 6-Item-Questionnaire

**Figure S2.** Scree plot showing the two-factor solution. For factor one and two, eigenvalues were >1, so a two-factor solution was confirmed as suitable.

| Item | Factor loadings | |
| --- | --- | --- |
|  | 1 | 2 |
| Kon_N_1 | .037 | **.467** |
| Kon_N_2 | **.751** | .033 |
| Kon_N_3 | **.753** | .123 |
| Kon_N_4 | **.447** | **.615** |
| Kon_N_5 | **.787** | .074 |
| Kon_N_6** | **.697** | **.370** |
| Kon_N_7** | **.866** | .048 |
| Kon_N_8** | **.668** | .371 |
| Kon_I_1 | **.364** | .298 |
| Kon_I_2 | .011 | **.641** |
| Kon_I_3** | **.387** | **.602** |
| Kon_I_4** | .054 | **.642** |
| Kon_I_5** | .210 | **.722** |
| Variance explained, % | 30.5 | 20.7 |
| Cumulative variance | 30.5 | 51.2 |

**Table S3.** Factor loadings showing the strength of the relationships between factors and items of the self-reported conformity scale. Bold indicates a factor loading of >.30. Factor loadings were extracted with the maximum likelihood method and the rotated with the varimax method.

**final 6-Item-Questionnaire
